# Cloning and Expression of Phytase appA Gene from *Shigella* sp. CD2 in *Pichia pastoris* and Comparison of Properties with Recombinant Enzyme Expressed in *E*. *coli*

**DOI:** 10.1371/journal.pone.0145745

**Published:** 2016-01-25

**Authors:** Moushree Pal Roy, Deepika Mazumdar, Subhabrata Dutta, Shyama Prasad Saha, Shilpi Ghosh

**Affiliations:** Department of Biotechnology, University of North Bengal, Siliguri, India; USDA-ARS, UNITED STATES

## Abstract

The phytase gene appA_S_ was isolated from *Shigella* sp. CD2 genomic library. The 3.8 kb DNA fragment contained 1299 bp open reading frame encoding 432 amino acid protein (AppA_S_) with 22 amino acid signal peptide at N-terminal and three sites of N-glycosylation. AppA_S_ contained the active site RHGXRXP and HDTN sequence motifs, which are conserved among histidine acid phosphatases. It showed maximum identity with phytase AppA of *Escherichia coli* and *Citrobacter braakii*. The appA_S_ was expressed in *Pichia pastoris* and *E*. *coli* to produce recombinant phytase rAppA_P_ and rAppA_E_, respectively. Purified glycosylated rAppA_P_ and nonglycosylated rAppA_E_ had specific activity of 967 and 2982 U mg^-1^, respectively. Both had pH optima of 5.5 and temperature optima of 60°C. Compared with rAppA_E_, rAppA_P_ was 13 and 17% less active at pH 3.5 and 7.5 and 11 and 18% less active at temperature 37 and 50°C, respectively; however, it was more active at higher incubation temperatures. Thermotolerance of rAppA_P_ was 33% greater at 60°C and 24% greater at 70°C, when compared with rAppA_E_. Both the recombinant enzymes showed high specificity to phytate and resistance to trypsin. To our knowledge, this is the first report on cloning and expression of phytase from *Shigella* sp.

## Introduction

Phytic acid (myo-inositol 1, 2, 3, 4, 5, 6-hexakis phosphate) is the major storage form of phosphorous in cereals, legumes, oil seeds and nuts [1]. Monogastric animals are incapable of digesting phytate phosphorous. Phytate also acts as an antinutritional agent, since it forms insoluble complexes with proteins and nutritionally important metal ions, such as calcium, copper and zinc and thus decreases nutrient bioavailability. The ingested phytate is largely excreted causing nutritional deficiencies and environmental pollution [1, 2].

Phytic acid is hydrolysed by phytase (myo-inositol hexakisphosphate hydrolase) to inorganic phosphate (Pi) and less phosphorylated myo-inositol derivatives [2, 3]. Phytase supplementation in animal feed increases the bioavailability of phosphorous in monogastric animals besides reducing the level of phosphorous output in their manure [4]. The enzyme is wide spread in nature, occurring in plants, animals and microorganisms. Phytases from these sources exhibit variations in structure and catalytic mechanism and consequently, have been categorized into cysteine phytases, histidine acid phosphatases (HAPs), β-propeller phytases and purple acid phosphatases [3]. Moreover, the ExPASy enzyme database (http://www.expasy.ch/enzyme/) classifies phytases into three different groups: 3-phytase (alternative name, 1-phytase; EC 3.1.3.8), 4-phytase (alternative name, 6-phytase; EC 3.1.3.26), and 5-phytase (EC 3.1.3.72). This classification is based on the carbon ring position where removal of phosphate groups from phytate is initiated [2–4].

A number of phytases have been characterized from various microorganisms such as *Aspergillus* species, *Citrobacter braakii*, *Obesumbacterium proteus*, *Bacillus subtilis*, *Escherichia coli*, *Pichia anomala*, *Erwinia carotovora* and *Yersinia intermedia* and corresponding genes have been isolated, cloned and expressed in different hosts [5–12].

Phytases belonging to HAP family have been used successfully as a feed additive. Although, the commercial production of phytase is currently focused on the fungal HAP from *Aspergillus* species, studies have suggested bacterial phytases as more promising because of their thermostability, higher substrate specificity, greater resistance to proteolysis and better catalytic efficiency. The substrate specificity property of the enzyme is highly desirable to prevent hydrolysis of other phosphate compounds so that they remain available for animal uptake [1, 2, 4].

The methylotrophic yeast *Pichia pastoris* has been successfully used as a host for heterologous gene expression, producing high level of recombinant proteins, including phytase. *P*. *pastoris* can grow in simple defined media, reach a very high cell density, and accumulates extremely high concentration of intra- or extracellular protein under the control of the *AOX1* promoter. In addition, *P*. *pastoris*, as a eukaryotic expression system, can carry out protein processing, folding, and posttranslational modifications [13, 14].

In our previous communication, we reported purification and characterization of phytase from *Shigella* sp. CD2 [15]. We herein report molecular cloning and sequencing of the phytase gene from *Shigella* sp. CD2 and its extracellular expression in *P*. *pastoris* strain GS115. The characteristic properties of the enzyme were compared with that expressed in *E*. *coli* strain BL21 (DE3).

## Materials and Methods

### Strains, plasmids and chemicals

The bacterial strain used in this study *Shigella* sp. CD2 (Accession no. FR745402) was isolated from wheat rhizosphere. The pUC18 vector, pGEM-T vector system, *E*. *coli* XL1 Blue and PCR reagents were purchased from Promega, USA. Restriction enzymes, Endo H deglycosylase and T4 DNA ligase were from New England Biolabs (Beverly, MA). *E*. *coli* BL21(DE3) and pET-20b(+) vector (Novagen, Madison, WI) and MagicMedia^TM^ *E*.*coli* Expression Medium (Invitrogen, San Diego,CA) were used for bacterial expression. The expression medium has two components, (a) Ready to use medium and (b) IPTG solution. For expression in eukaryotic system, *P*. *pastoris* GS115(*his4*) and pPIC9 expression vector were purchased from Invitrogen, San Diego, CA. Plasmid pPIC9 contains the promoter and terminator of the *P*. *pastoris AOX1* gene, the α-mating factor prepro-secretion signal from *S*. *cerevisiae* and the HIS4 auxotrophic selection marker for transforming *P*. *pastoris* GS115. Regeneration dextrose base (RDB), buffered glycerol-complex (BMGY), and buffered methanol-complex (BMMY) media were prepared according to the manual of the *Pichia* Expression kit (Invitrogen, San Diego, CA). All other chemicals and microbiological media were from Sigma Chemical Company, USA; E. Merck, Germany; and HiMedia Laboratory, India.

### Cloning of the phytase gene and nucleotide sequence analysis

Genomic DNA isolated from *Shigella* sp. CD2 [16] was partially digested with *Eco*RI to obtain 3 to 6 Kb fragments. The fragments were cloned in *Eco*RI site of pUC18 vector and transformed into *E*.*coli* XL1 Blue. The transformants were screened for phytase activity on LB-agar plates containing 100 μg mL^-1^ ampicillin and 1% sodium phytate. Phytase positive clones formed phytate clearance zone around the colony. The recombinant plasmid (pUCphy) was isolated from phytase positive clone with highest clearance zone; the 3.8kb insert in the plasmid was sequenced by using vector specific M13-pUC forward (5’- GTTTTCCCAGTCACGAC-3’) and reverse (5’-CAGGAAACAGCTATG-3’) primers and putative phytase encoding ORF was identified. The amino acid sequence encoded by the ORF was analyzed for the presence of signal peptide by SignalP 4.1 Server(http://www.cbs.dtu.dk/services/SignalP) [17] and for disulphide bond in the tertiary structure by using Softberry CYS_REC online services (www.softberry.com). Mature phytase gene without the signal sequence was amplified from pUCphy by using internal primers, PhyF (5’-ATGAATTCGCTCAGAGTGAGCCGGAG-3’ with 5’ *Eco*RI restriction site) and PhyR (5’GATGCGGCCGCCAAACTGCACGCCGGTATG-3’ with 5’ *Not*I site). The PCR product was cloned in pGEM-T vector following manufacturer’s instruction and sequenced using T7 and SP6 universal primers. Homology search in GenBank was done using the BLAST server (http://www.ncbi.nlm.nih.gov/BLAST) [18]. The amino acid sequence of the cloned gene was deduced and then aligned by ClustalW program (http://www.ebi.ac.uk/clustalW) [19]. The phylogenetic analysis of the protein was performed by neighbour joining method using MEGA 4 [20]. Bootstrap analysis was used to evaluate the tree topology of the neighbour joining data by performing 500 replicates. The tree was drawn to scale, with branch lengths in the same units as those of the evolutionary distances used to infer the phylogenetic tree. The recombinant pGEM-T vector harboring the phytase gene was named pGEMT-appA_S_.

### Construction of *P*. *pastoris* and *E*. *coli* expression plasmids and transformation

Two different plasmids were constructed for expression of appA_S_ in *P*. *pastoris* GS115 and *E*. *coli* BL21(DE3). For *P*. *pastoris* expression, the pGEMT-appA_S_ plasmid was cut with *EcoR*I and *Not*I. The resulting 1.2 kb DNA fragment was ligated into pPIC9 digested with *EcoR*I and *Not*I to generate pPIC9-appA_S_. The pPIC9-appA_S_ linearized with *Bsp*E1 was transformed into *P*. *pastoris* GS115 by the spheroplasting protocol according to the manual of the *Pichia* Expression kit (Invitrogen, San Diego, CA) and transformants were selected for ability to grow on histidine-deficient medium. The his^+^ transformants were further screened for Mut^+^ and Mut^S^ phenotypes. The integration of the expression cassette into the genome of *P*. *pastoris* GS115 was ascertained by PCR using the 5’ *AOX1* and 3’ *AOX1* primers. For expression in *E*. *coli*, the 1.2 kb fragment released from the pGEMT-appA_S_ plasmid was ligated into pET-20b(+) to generate the construct pET-20b(+)-appA_S_, which was transformed into *E*. *coli* BL21(DE3) and transformants were selected in presence of 100 μg mL^-1^ ampicillin.

### Expression of appA_S_ in *P*. *pastoris* GS115

The Mut^+^, pPIC9-appA_S_ transformed *P*. *pastoris* GS115 was inoculated into 10 mL of YPD (1% yeast extract, 2% peptone and 2% dextrose) and incubated overnight at 30°C and 300 rpm shaking. 1mL of starter culture was transferred to 100 mL of BMGY medium and grown at 30°C and 300 rpm shaking until culture reached an OD_600_ of 1. Cells were subsequently harvested by centrifugation at 2100×g for 5 min and used to inoculate 100 mL of BMMY medium containing 0.5% methanol as inducer. The culture was incubated at 30°C and 300 rpm shaking for 96 h and the induction was maintained by adding 0.5% (v/v) methanol at every 24 h intervals. Extracellular and periplasmic phytase activity and medium pH were monitored at every 12 h intervals. For isolation of extracellular fraction, the culture was centrifuged at 2100×g for 5 min and the cell free medium was concentrated and diafiltered by Vivaspin-20 (30 kDa cutoff) sample concentrator (GE Healthcare, UK). For periplasmic fraction isolation, cell pellet was submitted to 5 cycles of freezing (-20°C for 2 h) and thawing (28°C for 1 h), followed by extraction with 100 mM acetate buffer (pH 5.5) at 28°C in a rotatory shaker (100 rpm). The extracted sample served as periplasmic fraction. Induction of appA_S_ expression was determined by 12% SDS-PAGE analysis of the extracellular fraction. *P*. *pastoris* GS115 transformed with pPIC9 vector served as control. Recombinant protein produced by appA_S_ in *P*. *pastoris* GS115was named rAppA_P_.

### Expression of appA_S_ in *E*. *coli* BL21(DE3)

Expression of appA_S_ in *E*.*coli* BL21(DE3) was analysed by using MagicMedia^TM^ *E*.*coli* Expression Medium following manufacturer’s instruction. *E*. *coli* BL21 (DE3) cells transformed with pET-20b(+)-appA_S_ was grown overnight in LB medium at 37°C and 200 rpm shaking. The culture at 1% (v/v) was inoculated into the MagicMedia (19:1, ready to use medium: IPTG solution) and grown overnight at 37°C and 300 rpm shaking. The cells were then harvested by centrifugation at 11,200×g for 10 min, suspended in 50 mM acetate buffer (pH 5.5), disrupted by sonication and centrifuged. The supernatant and the pellet dissolved in 50 mM acetate buffer (pH 5.5) served as soluble and pellet fractions, respectively. Induction of appA_S_ expression in both the fractions was determined by 12% SDS-PAGE. Both the fractions were also checked for phytase activity. *E*. *coli* BL21 (DE3) transformed with pET-20b(+) vector was used as control. Recombinant protein produced by appA_S_ in *E*.*coli* BL21 (DE3) was named rAppA_E_.

### Protein estimation and SDS-PAGE analysis

Total protein concentration was determined by the dye binding assay of Bradford using bovine serum albumin (BSA) as standard [21]. SDS-PAGE analysis was performed with 12% polyacrylamide gel according to the method of Laemmli [22]. After electrophoresis, the gel was stained with CBB R-250 reagent (0.1% Coomassie Brilliant Blue R-250 in 10% acetic acid and 40% methanol) and then destained. Broad range pre-stained protein standards were used as markers.

### Purification of rAppA_E_ and rAppA_P_

Recombinant rAppA_P_ was purified from the cell free medium of pPIC9-appA_S_ transformed *P*. *pastoris* GS115 culture induced with methanol for 60 h. The concentrated and diafiltered cell-free medium was loaded on to CM-cellulose column and bound proteins were eluted by 50 mM acetate buffer (pH 5.5) with linear gradient of 0–0.5 M NaCl. The active fractions were pooled for subsequent studies. For purification of rAppA_E_, the IPTG induced culture of pET-20b(+)-appA_S_ transformed *E*.*coli* BL21 (DE3) was harvested by centrifugation at 11,200×g for 10 min. The cell pellet was suspended in 50 mM acetate buffer (pH 5.5), disrupted by sonication and centrifuged. The supernatant was loaded onto a Ni- Sepharose Fast Flow column (2 x 5 cm, GE Healthcare, UK) pre-equilibrated with 50 mM acetate buffer (pH 5.5) containing 10 mM imidazole. The bound proteins were eluted with 50 mM acetate buffer (pH 5.5) containing 100 mM imidazole. Fractions with phytase activity were pooled for subsequent studies.

### Determination of phytase activity

Phytase activity was determined as described previously [15]. The reaction mixture in a final volume of 2 mL contained, acetate buffer (pH 5.5), 100 mM; sodium phytate, 2 mM; and 100 μL enzyme preparation. The reaction was carried out at 37°C for 30 min followed by termination of reaction by adding 2 mL of 10% trichloroacetic acid. The released Pi was measured spectrophotometrically by adding 2 mL of ammonium molybdate (0.5%), sulphuric acid (5 N) and ascorbic acid (2%) solution. One unit (U) of phytase activity represents 1 μmol of Pi released min^-1^ under assay conditions.

### Characterization of rAppA_E_ and rAppA_P_

The pH optima was determined by measuring enzymatic activity at pH 2.5–8.5 in the following buffers (50 mM): glycine-HCl (pH 2.5 and 3.5), sodium acetate (pH 4.5 and 5.5), and Tris-HCl (pH 6.5, 7.5 and 8.5). The optimum temperature for activity was determined at temperatures ranging from 10 to 80°C. Thermostability of the enzyme was determined by preincubating the purified enzyme at 10 to 80°C for 30 min followed by measuring phytase activity under standard conditions. To study the effect of metal ions and salts (2 mM), phytase activity was monitored in presence of CaCl_2_, MnSO_4_, MgSO_4_, FeSO_4_, ZnSO_4_, CuSO_4_ and EDTA. To determine the susceptibility to digestive proteases, the 50 U of purified rAppA_E_ or rAppA_P_ was preincubated with pepsin and trypsin (30 U, Sigma) at 37°C and phytase activity was monitored 30 min later.

Substrate specificity of the enzyme was determined by replacing sodium phytate in the standard reaction mixture of various pH (pH 4.5–7.5) with an equal concentration (2 mM) of either of phosphorylated compounds, such as p-nitrophenyl phosphate (pNPP), ATP, ADP, disodium pyrophosphate (dSPP), D-glucose-6-phosphate (G6P) and D-fructose-6-phosphate (F6P). *K*_*m*_ for phytate was determined using the Lineweaver-Burk plot. *K*_*cat*_ values for both the enzymes were also determined.

### Deglycosylation

The deglycosylation of rAppA_P_ was carried out using Endo H deglycosylase (New England Biolabs) following manufacturer’s instruction. The reaction mix containing, 50 U of purified rAppA_P_, 600 μL of 50 mM Tris buffer (pH 7.0) and 10 U of Endo H in final volume of 1 mL, was incubated at 37°C for 2 h. N-glycosylation was determined by assessing the migration shift of Endo H treated rAppA_P_ in 12% SDS-PAGE.

### Western blot analysis

For immunoblot analysis, purified rAppA_E_ and deglycosylated rAppA_P_ proteins separated by 12% SDS-PAGE, were transferred to polyvinylidenedifluoride (PVDF) membrane by semi-dry method using Electroblotting apparatus (Atto, Japan). Purified rabbit antibody raised against *E*. *coli* phytase, diluted 1:1000 prior to application, was the primary antibody. The reacted polypeptide was visualised with a secondary antibody, goat anti-rabbit IgG-alkaline phosphatase conjugate using colorimetric based nitroblue tetrazolium chloride and 5-bromo-4-chloro-3-indolylphosphate-p-toluidine (NBT/BCIP) detection kit (Invitrogen, USA). Broad range prestained standards were used as markers.

## Results

### Isolation of gene encoding phytase from the genomic library

**Fig 1:**
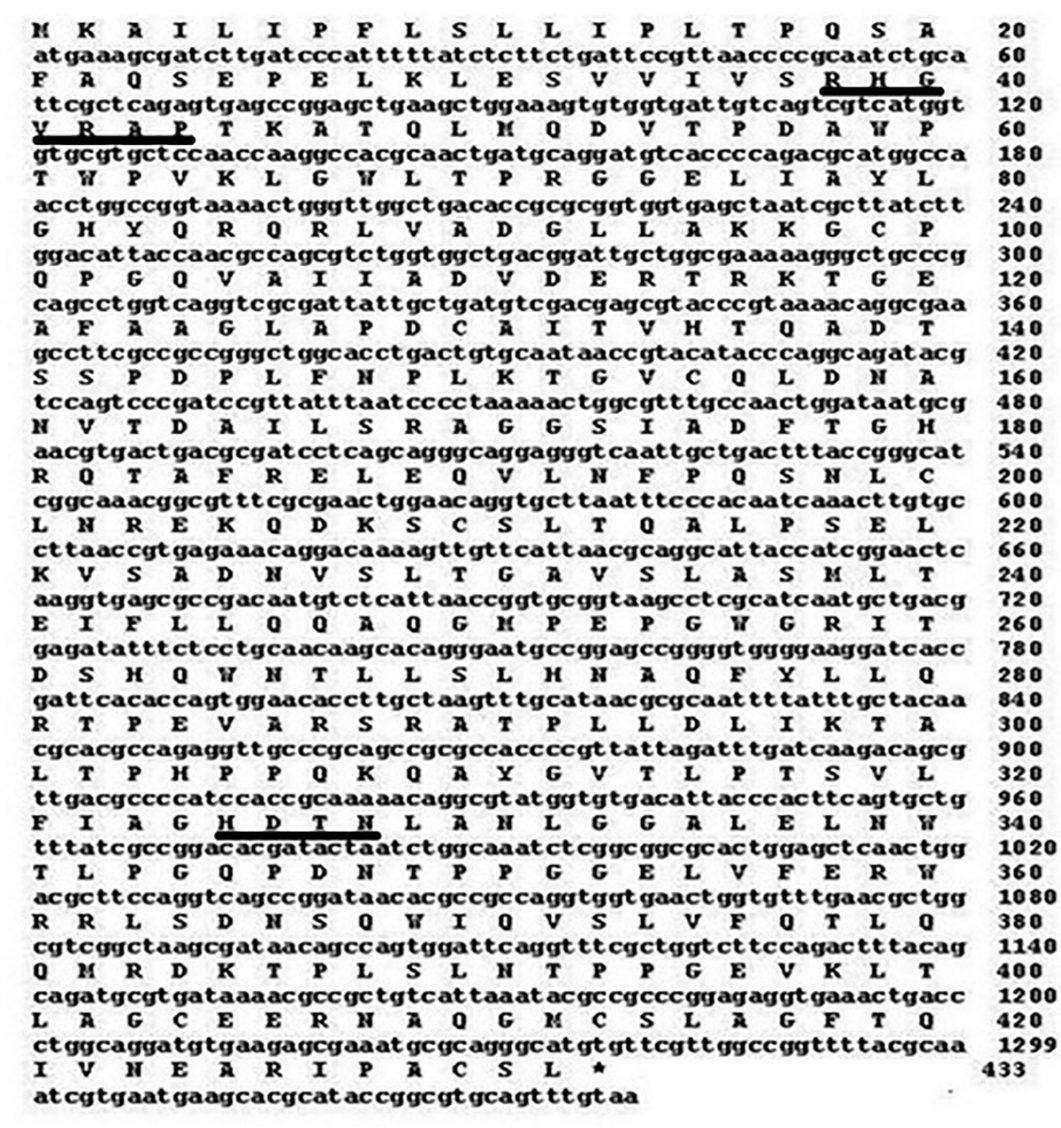
Nucleotide (1–1299) and deduced amino acid sequences (432) of the putative phytase gene appA_S_, from *Shigella* sp. CD2. The conserved HAP family active site motifs are underlined. Stop codon is shown by asterisk.

For cloning of phytase gene, a size selected genomic library of *Shigella* sp. CD2 was constructed in pUC18 vector using *Eco*RI digested genomic DNA. The library was screened for phytase activity based on formation of clearance zone in phytate-agar medium. Among the six phytase positive clones, one with highest phytate clearance zone and phytase activity in the cell lysate was selected. The clone harbouring plasmid pUC18-phy had DNA insert of 3.8 kb. Sequence analysis of the insert indicated presence of an open reading frame (ORF) of 1299 bp, encoding a protein of 432 amino acids (Fig 1).

### Sequence and phylogenetic analysis

**Fig 2:**
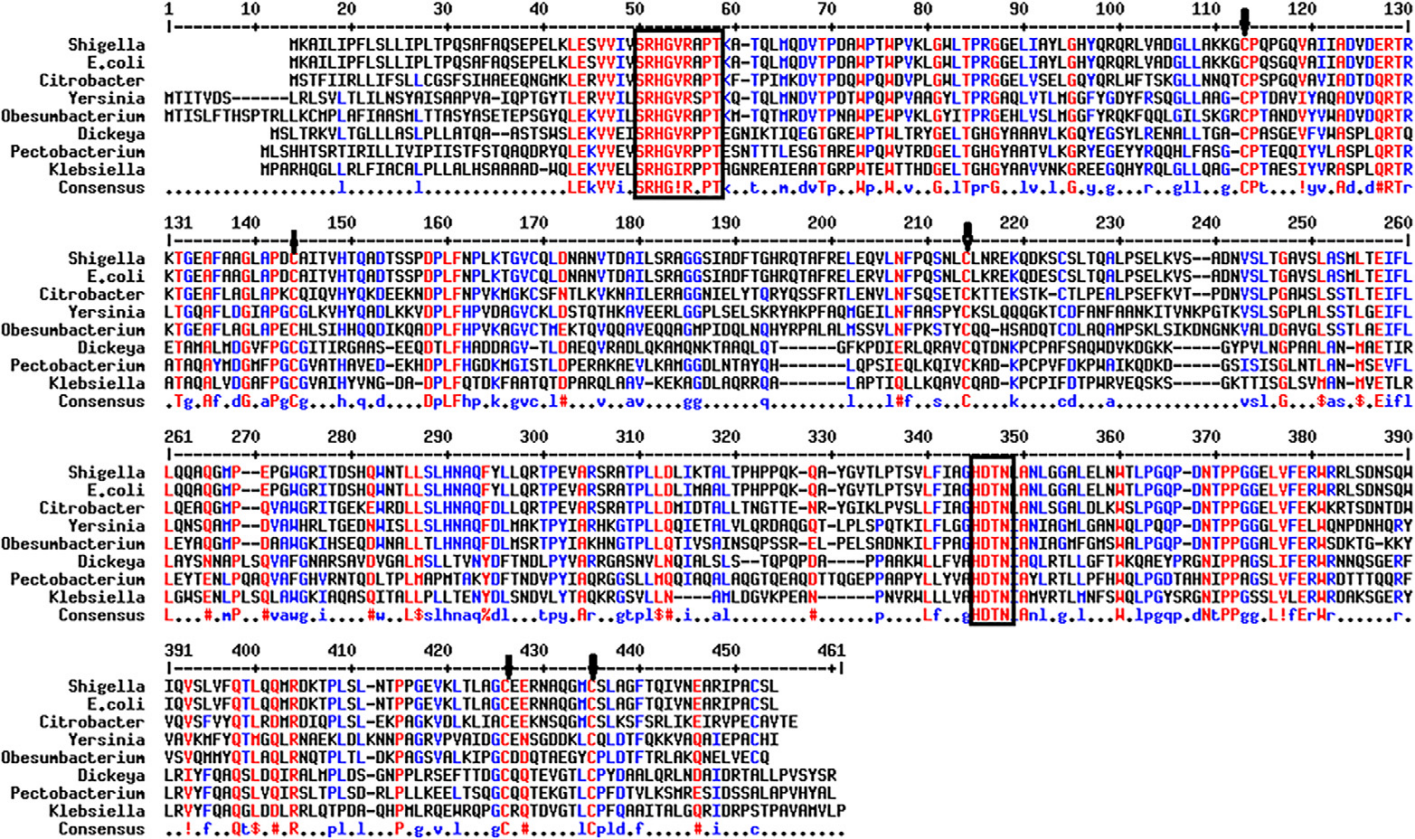
Multiple alignment of homologs of the *Shigella* sp. CD2 phytase AppA_S_. Conserved active site motifs are boxed and conserved cysteine residues are shown by arrows. The source and GenBank Accession Nos. of proteins are: *Shigella* sp. CD2, CCA94903; *Escherichia coli* AppA, EDX38944; *Dickeya paradisiaca*, ABW76125; *Klebsiella pneumoniae* ASR1, AAM23271; *Yersinia intermedia*, ABI95370; *Citrobacter braakii*, AAS45884; *Obesumbacterium proteus*, AAQ90419; *Pectobacterium carotovorum* subsp. *carotovorum*, ABY76184.

**Fig 3:**
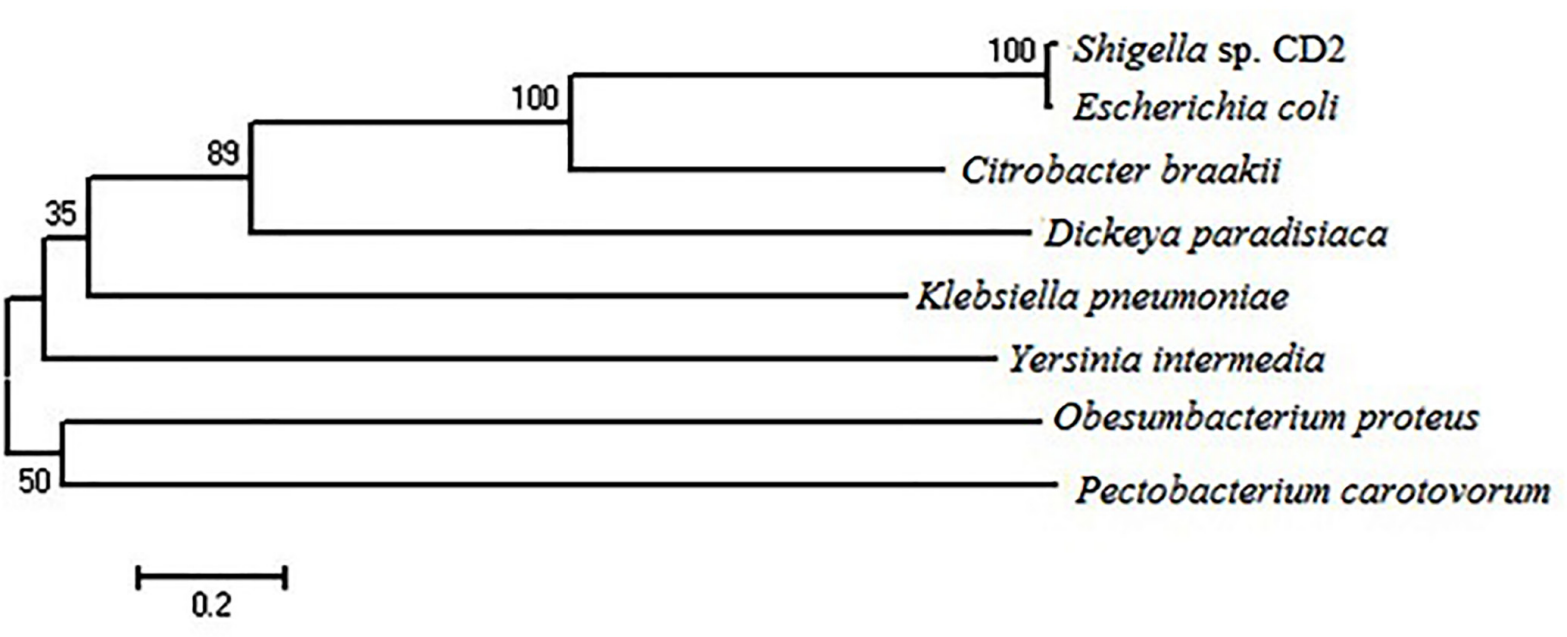
Phylogenetic tree of homologs of the *Shigella* sp. CD2 phytase AppA_S_. The bar represents 2 substitutions per 10 amino acids. GenBank Accession Nos. are as in Fig 2 legend.

Homology analysis of deduced amino acid sequence by BLAST program revealed 98 and 62% similarity with AppA phytase of *E*. *coli* and *C*. *braakii*, respectively. Hence, *Shigella* sp. CD2 phytase ORF was named as appA_S_ and the encoded protein as AppA_S_. The nucleotide sequence was deposited in the GenBank under accession number FR865899. AppA_S_ contained three potential sites of N-glycosylation, a putative signal peptide of 22 amino acids at N-terminal end and 8 cysteine residues among which 99–130, 200–210, 404–413 were the most possible disulphide bond pairs. The calculated molecular mass of the protein with and without the signal sequence were about 47 and 45 kDa, respectively. Alignment of AppA_S_ with other enteric bacterial phytases in the GenBank using ClustalW program showed presence of N-terminal RHGXRXP motif, C-terminal HDTN motif and five conserved cysteine residues. AppA_S_ and *E*. *coli* AppA differed in sequence at six positions; AppA_S_ contained P, Q, N, K, K, T in place of S, R, K, E, M, A in *E*. *coli* AppA at positions 102, 190, 202, 208, 298, 299, respectively (Fig 2). A phylogenetic tree was constructed based on the alignment using the neighbour joining method. The topology of the phylogram also confirmed AppA_S_ to be closely related to AppA phytase of *E*. *coli* and *C*. *braakii* (Fig 3).

### Expression of appA_S_ in *P*. *pastoris* G115

The appA_S_ was cloned in *Eco*RI and *Not*I sites of *P*. *pastoris* expression vector pPIC9. The recombinant plasmid pPIC9-appA_S_ carried the appA_S_-expression cassette consisting of 1.2 kb appA_S_ gene in frame with *S*. *cerevisiae* α-factor secretion signal, flanked by *AOX1* promoter and terminator sequences. Transformation of linearized pPIC9-appA_S_ into *P*. *pastoris* GS115 gave about 20 his^+^ transformants. The integration of appA_S_-expression cassette into the host genome was ascertained by PCR using 5’ and 3’ *AOX1* primers. PCR amplification products of about 0.5kb and 1.7 kb in pPIC9 transformed and pPIC9-appA_S_ transformed *P*. *pastoris* GS115, respectively, indicated the integration of appA_S_-expression cassette into the genome of the later.

**Fig 4:**
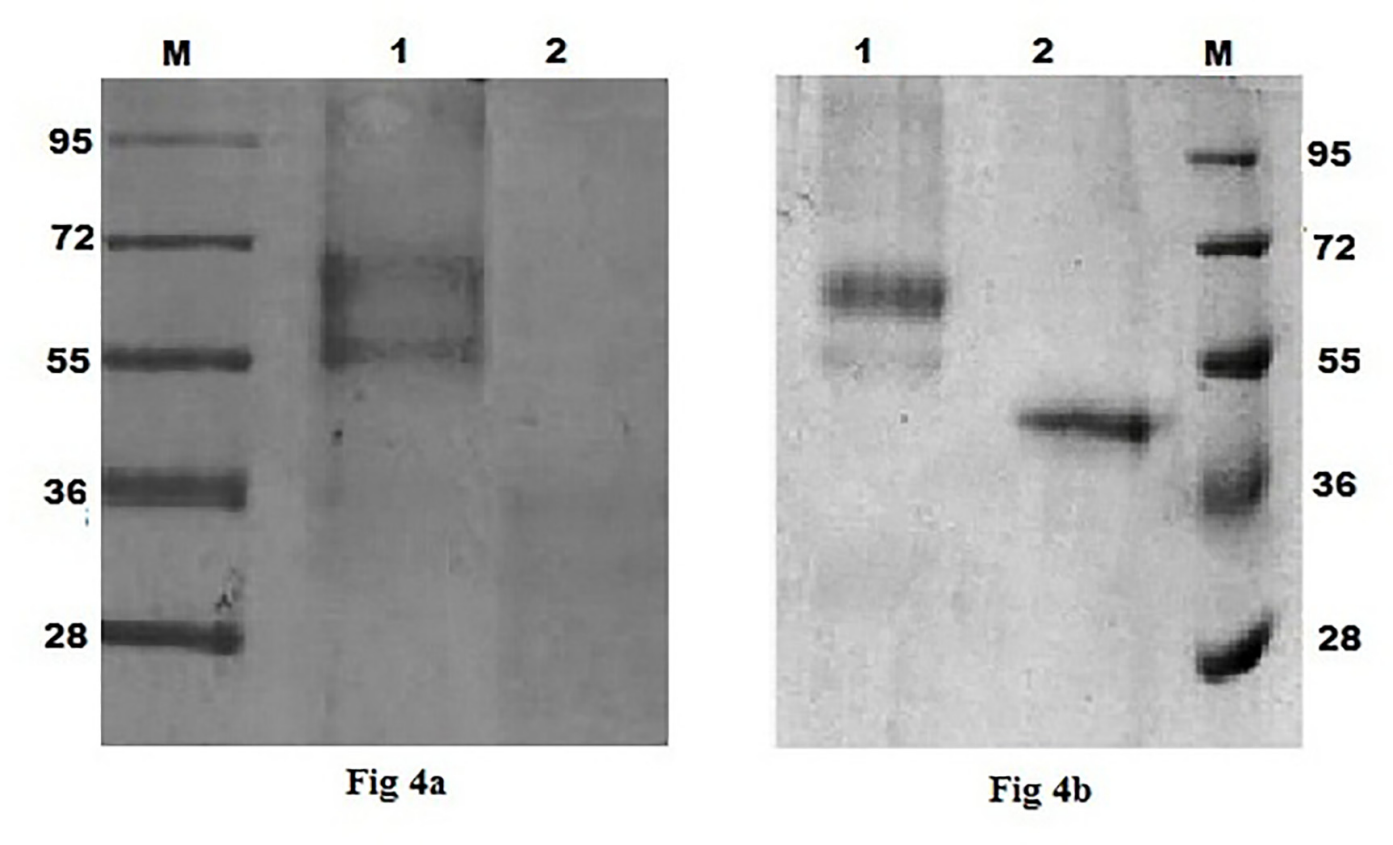
(a) SDS-PAGE analysis of rAppA_P_ expressed in *P*. *pastoris* GS115. Lane, M-molecular weight markers, 1-extracellular fraction of *P*. *pastoris* GS115 transformed with pPIC9-appA_S_, 2- extracellular fraction of *P*. *pastoris* GS115 transformed with pPIC9. (b) SDS-PAGE analysis of glycosylated and deglycosylated rAppA_P_. Lane, 1- glycosylated rAppA_P_, 2- deglycosylated rAppA_P_, M- molecular weight markers.

The pPIC9-appA_S_ transformed *P*. *pastoris* GS115 colonies were screened for Mut phenotypes, and for extracellular and periplasmic phytase activity. A Mut^+^ colony with highest extracellular phytase activity was selected for shake flask expression. At 60 h of methanol induction, the selected transformant showed maximum extracellular recombinant phytase (rAppA_P_) production of 62 U mL^-1^ with specific activity 477 U mg^-1^ and an extracellular protein concentration of 0.13 mg mL^-1^. SDS-PAGE analysis of concentrated and diafiltered cell-free extract showed two protein bands of approximate molecular mass 59 and 65 kDa (Fig 4A). Deglycosylation of rAppA_P_ by Endo H deglycosylase resulted in single band of apparent molecular mass 45kDa (Fig 4B).

### Expression of appA_S_ in *E*. *coli* BL21(DE3)

**Fig 5:**
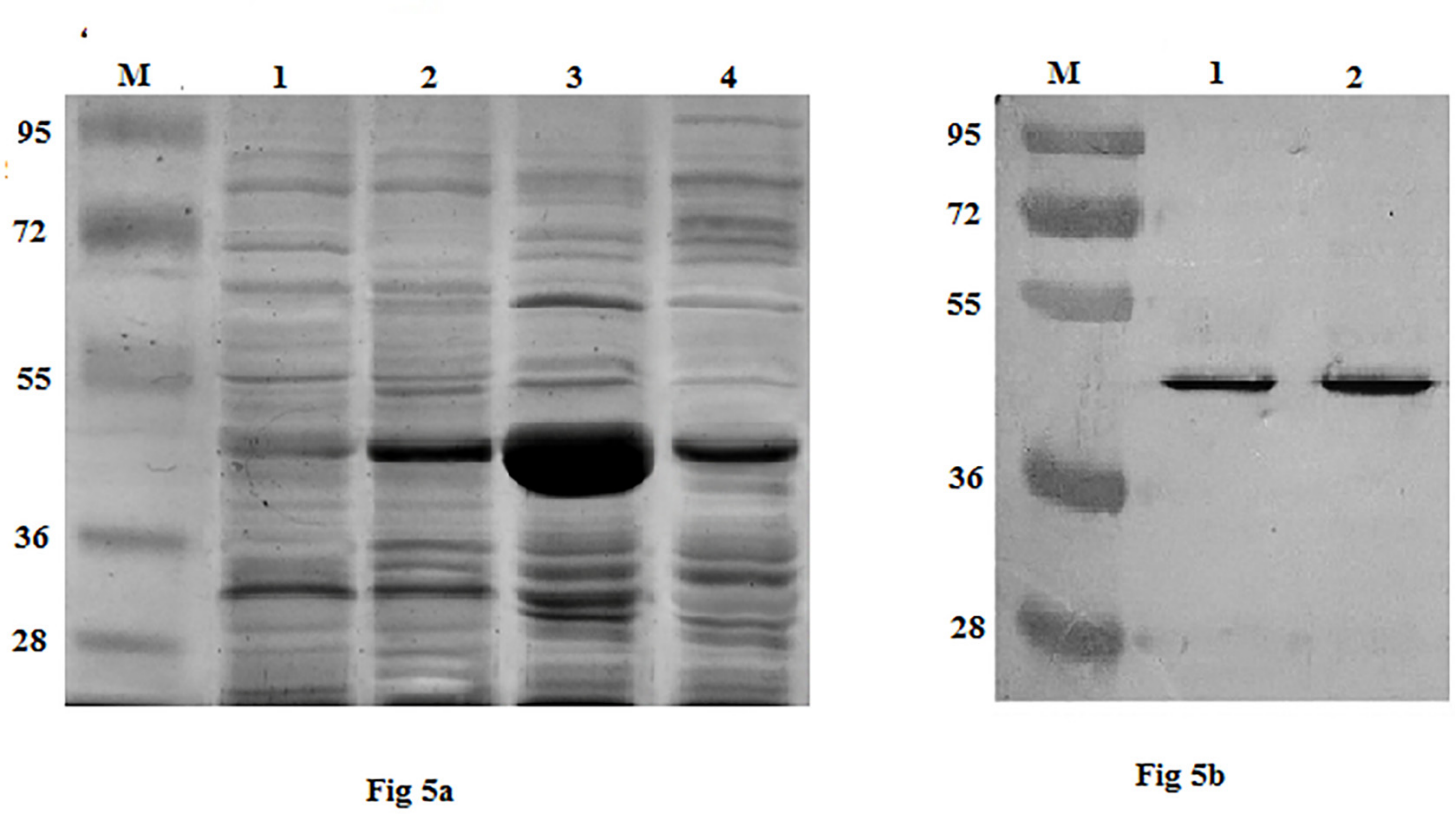
(a) SDS-PAGE analysis of rAppA_E_ expressed in *E*. *coli* BL21(DE3). Lane, M-molecular weight markers, 1- soluble fraction of induced BL21 transformed with pET20b(+), 2-pellet fraction of induced BL21 transformed with pET20b(+), 3- soluble fraction of induced BL21 transformed with pET-appA_S_, 4- pellet fraction of induced BL21 transformed with pET-appA_S_. (b) Western blot analysis. Lane, M- Molecular weight marker, 1- purified rAppA_E_, 2-purified and deglycosylated rAppA_P_.

The mature appA_S_ was cloned into *E*. *coli* expression vector pET-20b(+) and the recombinant plasmid pET-20b(+)-appA_S_ was transformed into *E*. *coli* BL21(DE3). The transformant was induced in MagicMedia supplemented with IPTG and after overnight induction cells were disrupted by sonication. Recombinant phytase (rAppA_E_) overexpression in the soluble and pellet fractions of sonicated cells was analyzed by SDS-PAGE. As shown in the results of Fig 5A, the soluble fraction of the induced cell exhibited protein overexpression band of approximately 45kDa, which agrees with the predicted molecular weight deduced from the amino acid sequence of AppA_S_. Phytase activity in the soluble fraction was 176 U mL^-1^ (specific activity 568 U mg^-1^), whereas negligible activity was detected in the pellet fraction. The results thus indicate a correlation of rAppA_E_ overexpression with phytase activity. Western blot analysis of rAppA_E_ and deglycosylated rAppA_P_ using rabbit polyclonal antibody against *E*. *coli* AppA further demonstrated that the specific band with apparent molecular mass of 45 kDa was recombinant phytase (Fig 5B).

### Purification and properties of rAppA_E_ and rAppA_P_

**Fig 6:**
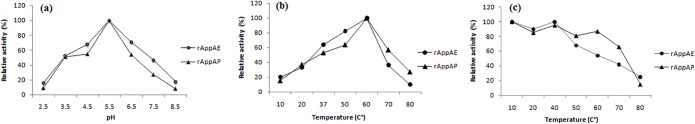
Characterization of purified rAppA_E_ and rAppA_P._ (a) pH profile (b) Temperature profile and (c) Thermal stability. Results of phytase activity represent the mean of three independent values.

Recombinant rAppA_P_ was purified by cation exchange chromatography of diafiltered extracellular fraction of methanol induced *P*. *pastoris* GS115 culture transformed with pPIC9-appA_S_ and rAppA_E_ was purified from the soluble fraction of pET-20b(+)-appA_S_ transformed *E*. *coli* BL21 (DE3) using Ni-Sepharose Fast Flow affinity chromatography. Purified rAppA_P_ and rAppA_E_ had specific activities of 967 and 2982 U mg^-1^, with recovery of 75 and 83%, respectively. The results of biochemical properties of rAppA_P_ and rAppA_E_ are shown in Table 1. Compared with the glycosylated rAppA_P_, the nonglycosylated rAppA_E_ was more active at pH 3.5–7.5. Both the enzymes had more than 50% activity in the pH range 3.5 to 6.5 with pH optima at 5.5 (Fig 6A). Both rAppA_E_ and rAppA_P_ had temperature optima of 60°C. Compared with rAppA_P_, rAppA_E_ had 11 and 18% greater relative activity at 37 and 50°C, respectively, whereas at higher incubation temperature rAppA_P_ was more active than rAppA_E_ (Fig 6B). For determination of thermal stability, the purified rAppA_E_ or rAppA_P_ were pre-incubated at 10 to 80°C for 30 min and then assayed for enzymatic activity. Although, the two enzymes didn’t differ in their thermostability in the temperature range 10 to 50°C, rAppA_P_ was more thermotolerant at higher temperature. Consequently, at 60 and 70°C rAppA_P_ had 33 and 24% higher activity in comparison to rAppA_E_, respectively (Fig 6C). *K*_*m*_ values for phytate as determined by Lineweaver-Burk plot were 0.18 and 0.22 mM for rAppA_E_ and rAppA_P_, respectively (Table 1). The *K*_*cat*_ value for rAppA_E_ was 2.23×10^3^ sec^-1^and for rAppA_P_ was 0.72×10^3^ sec^-1^.

**Table 1:** Properties of rAppA_E_ and rAppA_P_.

Properties	Results	
	rAppA_E_	rAppA_P_
*Substrate specificity (Sodium phytate)	100%	100%
*K*_*m*_ for phytate (mM)	0.18	0.22
*V*_*max*_ (μmol min^-1^)	149.1	48.35
*K*_*cat*_ (Sec^-1^)	2.23×10^3^	0.72×10^3^
*K*_*cat*_ */K*_*m*_ (Sec^-1^ mM^-1^)	12.43×10^3^	3.23×10^3^
Specific activity of purified enzyme (U mg^-1^ protein, 37°C)	2982	967
Temperature optima (°C)	60	60
pH optima	5.5	5.5
^$^Thermostability (%)	100	100
^#^Activity in presence of trypsin	70%	65%
^#^Activity in presence of pepsin	55%	50%
Activity in presence of metal ions (20 mM): Ca^2+^	130%	105%
Mg^2+^	125%	110%
Mn^2+^	109%	102%

*Activity in presence of ATP, ADP, pNPP, dSPP, G6P, F6P was negligible.

^$^Activity after pre-incubation of enzyme at 40°C for 30 min.

# Recombinant enzyme (50 U) was pre-incubated with pepsin or trypsin for 60 min followed by determination of phytase activity.

Both rAppA_E_ and rAppA_P_ were highly specific to the substrate, sodium phytate. Activity with either of phosphorylated substrates, such as ATP, ADP, pNPP, dSPP, G6P or F6P was negligible. The relative phytase activities of rAppA_E_ and rAppA_P_ were enhanced up to 130% in presence of Ca^2+^, Mg^2+^ and Mn^2+^, whereas Cu^2+^, Fe^2+^, Zn^2+^ or EDTA showed inhibitory effect. To determine the protease resistance the purified recombinant phytases (50 U) were pre-incubated separately with 30U of either pepsin or trypsin at 37°C. The rAppA_E_ and rAppA_P_ retained 70 and 65% activity on treatment with trypsin, and 55 and 50% of activity on treatment with pepsin, respectively, indicating greater resistance to trypsin.

## Discussion

The phytase structural gene (appA_S_) from *Shigella* sp. CD2 had an ORF of 1299 bp encoding 432 amino acid protein (AppA_S_) containing N-terminal 22 amino acid signal peptide, three probable disulphide bridges and three sites of N-glycosylation. Presence of the signal peptide and disulphide bridges indicates the periplasmic localization of the native protein. AppA_S_ showed significantly high homology with AppA phytase of *E*. *coli* and *C*. *braakii* suggesting that the proteins may have similar structure and mechanism of action. Moreover, all these AppA phytases form a separate branch in phylogenetic tree. As in the present study, phytase AppA from *C*. *braakii* was more closely related to the *E*. *coli* AppA than to other phytases [6]. AppA_S_ contained the conserved N-terminal RHGXRXP and C-terminal HD active site motifs, and six conserved cysteine residues, which are characteristics of phytase belonging to the HAP family [23]. Till date, seven genera of family enterobacteriaceae have been reported to produce phytase, and relevant genes have been cloned and all of them belong to HAP family [6, 7, 11, 12, 23].

The expression of enzyme as secreted protein is one of the useful and important characteristics for its economical production in industry. *P*. *pastoris* has been successfully used as host organism for extracellular production of recombinant proteins at high level, including phytase [2, 5, 6, 10, 11]. Phytase appA_S_ was expressed in *P*. *pastoris* to produce rAppA_P_ as extracellular protein with highest activity (62 U mL^-1^) at 60 h of methanol induction, with specific activity of 477 U mg^-1^. The rAppA_P_ activity is higher than that of *phy*C gene encoding neutral phytase expressed in *P*. *pastoris* (12.5 U mL^-1^) [2]. However, the yield is lower than that of AppA phytase of *C*. *braakii* (197 U mL^-1^) and *E*. *coli* (112.50 U mL^-1^) [6, 9]. The lower activity of rAppA_P_ might be due to observed increase in the medium pH above 7 during cultivation of *P*. *pastoris*. This could be confirmed by significant decrease in activity of purified AppA_P_ at pH >7 (Fig 6A). The expression level and activity of rAppA_P_ could be increased further by optimization of bioprocess and control of medium pH at <7. Moreover, the reduced phytase activity could also be due to the variation in codon usage between *Shigella* sp. and *P*. *pastoris*. Previous studies have shown the effect of codon bias on expression and activity of recombinant phytase and other enzymes [14, 24]. Xiong *et al*. used *P*. *pastoris* preferred codons and modified signal sequences to improve the expression of heterologous phytase from *Peniophora lycii* by 13.6 fold [14]. Similarly, extracellular expression of *phy*C gene from *B*. *subtilis* WHNB02 in *P*. *pastoris* yielded 2.40 U mL^-1^ phytase. Synthesis of *phy*C according to *P*. *pastoris* codon usage without altering the protein sequence enhanced activity by about 8 folds to 18.50 U mL^-1^ [24]. The recombinant rAppA_P_ was expressed as multiple proteins of higher molecular weights, which on deglycosylation produced protein of about 45 kDa, similar to that of rAppA_E_, indicating post translational glycosylation of the recombinant protein in *Pichia* system (Fig 4A and 4B). As in the present study, SDS-PAGE analysis of recombinant AppA from *E*. *coli* expressed in *P*. *pastoris* appeared as diffused band of molecular size 55 kDa, however, a sharp band was observed after the purified phytase was deglycosylated [9]. Similarly, AppA from *C*. *braakii* expressed in *Saccharomyces cerevisiae* migrated as a broad diffusion band (110–160 kDa) in SDS-PAGE gel due to extensive N-linked glycosylation, while the same protein expressed in *E*. *coli* had molecular size of 49 kDa [6].

To examine the effect of glycosylation on enzymatic properties of rAppA_P_, appA_S_ was also expressed in *E*. *coli* to produce rAppA_E_. The periplasmic signal sequence was removed for targeting the enzyme to the intracellular space in order to avoid the possibility of contamination of recombinant enzyme preparation with two native periplasmic AppA phytases in the host cell [25]. Phytase activity of nonglycosylated rAppA_E_ was 176 U mL^-1^ (specific activity 568 U mg^-1^). The rAppA_E_ activity is significantly higher than that of *phy*A gene of *O*. *proteus* (9.6 U mg^-1^) and *app*A gene of *E*. *coli* (17.1 U mg^-1^) expressed in *E*. *coli* as intracellular proteins [7]. Most of the other studies on expression of recombinant phytase in *E*. *coli* have shown accumulation of phytase as inclusion body in the cell [6, 26].

The purified rAppA_P_ and rAppA_E_ had specific activities of 967 and 2982 U mg^-1^, respectively. The difference in glycosylation between the two enzymes partially affected their biochemical properties. Both the recombinant enzymes had pH optima of 5.5 and more than 50% of activity was maintained between pH 3.5 to 6.5. The pH optimum of most of the enterobacterial phytase AppA is in the range of 4.5 to 5.5. The enzyme from *E*. *coli*, *O*. *proteus*, *C*. *braakii*, *Y*. *intermedia*, and *E*. *carotovora* showed optimum pH of 4.5, 4.9, 5.0, 4.5 and 5.5, respectively [6,7, 9,11,12]. Although rAppA_P_ and rAppA_E_ shared the same optimal temperature of 60°C, the former was more active at 70 and 80°C. As in the present study, the temperature optima of other reported bacterial AppA phytases were in the range of 40–65°C [7, 9,11,12]. Glycosylated rAppA_P_ had improved thermotolerance, especially at higher temperatures of 60 and 70°C over that of rAppA_E_. The *K*_*m*_ values of 0.18 mM for rAppA_E_ and 0.22 mM for rAppA_P_ are less than that of the phytases from *O*. *proteus*(0.34 mM), *E*. *coli* (0.55 mM), *E*. *carotovora* (0.25 mM), *K*. *pneumoniae* (0.28 mM) [7, 9,12, 27], but higher than that of the phytase from *Y*. *intermedia* (0.125 mM) [11]. The catalytic efficiency of rAppA_E_ was found to be much higher than that of rAppA_P_ as reflected by their *K*_*cat*_ values.

Glycosylaion is one of the most important post translational modifications that affects protein function and properties. Previous studies have shown the influence of N-glycosylation on biochemical properties of proteins, such as molecular mass, isoelectric point, surface charge distribution and thermotolerance [3, 28]. As in the present study, increased level of glycosylation of phytase from *A*. *fumigatus* expressed in *P*. *pastoris* improved the thermotolerance of the protein over the deglycosylated form [28]. Similarly, phytase from *C*. *braakii* expressed in *S*. *cerevisiae* retained 50% higher activity upon heat treatment at 70°C for 30 min as compared to *E*. *coli* expressed protein [6]. Although there are very few studies on effect of glycosylation on *K*_*m*_, recently Yao *et al*. reported an alteration in *K*_*m*_ of recombinant *E*. *coli* AppA phytase on enhancement of glycosylation. The *K*_*m*_ values for WT, Q258N mutant and Q258N/Q349N mutant were 0.48, 0.53 and 0.43 mM, respectively [29]. Phytase in the present study was highly specific to the substrate phytate as observed for AppA phytase from *E*. *carotovora* and *Y*. *intermedia* [11,12], whereas phytase from *E*. *coli* and *O*. *proteus* also cleaved phosphorus-containing organic compounds other than phytate at a slower rate [7, 9]. In contrast, phytases from *Aspergillus fumigatus* and *Klebsiella pneumoniae* showed broad specificity for phosphorylated substrates but relatively low specificity for phytate [27].

In conclusion, phytase AppA_S_ expressed in *P*. *pastoris* (rAppA_P_) had biochemical properties similar to that expressed in *E*. *coli* (rAppA_E_), except for thermalstability. The enzyme has several advantageous properties, like substrate specificity, protease resistance, optimal activity at acidic pH and physiological temperature. Phytase AppA from *Shigella* sp. CD2 displayed 40–70% activity in the pH range 3.5 to 6.5, which can facilitate phytate degradation in salivary gland (pH 5.0–7.0), stomach (fed state pH 6.5, reducing to 3.5–4.5 upon stimulation of acid secretion) and upper part of duodenum (pH 4.0–6.0). Hence, the enzyme can be used as feed additive for improving the utilization of phytate phosphorus by monogastric animals like, swine, poultry and farm animals. Though production of rAppA_P_ as secreted protein is advantageous for industry, its economical production requires improving its expression by using *P*. *pastoris*-preferred codons and optimization of bioprocess and scaling up when the cells are grown in a fermenter. Hence, there is a potential to increase the expression level even further, which is being pursued in the laboratory.
